# A Systematic Review of Mixed Studies Exploring the Effects of Probiotics on Gut-Microbiome to Modulate Therapy in Children With Autism Spectrum Disorder

**DOI:** 10.7759/cureus.32313

**Published:** 2022-12-08

**Authors:** Maithily Patel, Lakshmi M Atluri, Natalie A Gonzalez, Navya Sakhamuri, Sreekartthik Athiyaman, Bhawna Randhi, Sai Dheeraj Gutlapalli, Jingxiong Pu, Maheen F Zaidi, Safeera Khan

**Affiliations:** 1 Family Medicine, California Institute of Behavioral Neurosciences & Psychology, Fairfield, USA; 2 General Surgery, California Institute of Behavioral Neurosciences & Psychology, Fairfield, USA; 3 Pediatrics, Medical University of Graz, Graz, AUT; 4 Pediatrics, California Institute of Behavioral Neurosciences & Psychology, Fairfield, USA; 5 Internal Medicine, California Institute of Behavioral Neurosciences & Psychology, Fairfield, USA; 6 Internal Medicine, California Institute of Behavioral Neurosciences & Psychology, Fairfield, CA, USA; 7 Medicine, California Institute of Behavioral Neurosciences & Psychology, Fairfield, USA; 8 Psychiatry and Behavioral Sciences, California Institute of Behavioral Neurosciences & Psychology, Fairfield, USA; 9 Research, California Institute of Behavioral Neurosciences & Psychology, Fairfield, USA

**Keywords:** autism spectrum disorder, gut microbiome, probiotics, autistic, microflora, microbiota, gut flora, lactobacillus

## Abstract

Autism spectrum disorder(ASD) is a complex neurodevelopmental disorder characterized by social deficits, repetitive typical behaviors, insistence on the same routines, and communication impairments. The prevalence of ASD has increased in the past decade. While we are aware that there is no cure for ASD, attempts are being made to reduce its symptoms and improve the learning, overall growth, and well-being of ASD patients. Gastrointestinal (GI) symptoms are frequent occurrences in patients with ASD, but the underlying mechanisms are unknown. Recent studies show that the microbiota-gut-brain axis is the key modulator of neuropsychiatric health. Although fecal transplants have shown positive outcomes in treating dysbiosis and symptoms of autism, lifestyle modifications such as dietary intervention will prevent and treat this disorder without causing major adverse effects. Probiotics enhance the microbiome to provide necessary metabolites, which help in gut permeability, cognitive function, and immunity. In some studies, children with increased GI symptoms have also shown increased behavioral disturbances. In this study, a systematic review of mixed studies is conducted to obtain more robust and conclusive results. We included randomized controlled studies with larger sample sizes and specifications on probiotics.

## Introduction and background

The Centers for Disease Control and Prevention’s (CDC) Autism and Developmental Disabilities Monitoring Network mentions that one in 68 children has autism spectrum disorder (ASD) [1]. It is more common in men, and it has been seen in a recent meta-analysis that the male-to-female ratio is nearly 3:1 compared to a previous report of 4:1 [2]. However, the Diagnostic and Statistical Manual of Mental Disorders Fifth Edition (DSM-5) criteria notably were not used in that study [2]. The study also showed that females who meet the criteria for ASD are at a higher risk of not receiving a clinical diagnosis [2]. The etiology of ASD includes factors such as epigenetics, genetics, and the environment. In total, 16 newly identified genes have been correlated with ASD, leading to new potential mechanisms such as ion transport and cellular cytoskeletal structure [3]. In 2014, the estimated annual cost of childhood ASD in the United Kingdom was found to be £3.4 billion and in the United States was US$66 billion [4]. In a recent Scottish report, the annual cost of ASD in Scotland was reported to be almost £2.3 billion, of which 93% was accounted for by adults and 7% by children [4]. Children with ASD often have dysbiosis with unfavorable effects on both the gastrointestinal tract and psychological symptoms. The treatment options include both pharmacological and non-pharmacological options. Dietary exclusions and probiotic supplements have, thus, been investigated.

In a study, Rose et al. [5] compared the microbiota and immune responses in children with ASD with and without gastrointestinal (GI) symptoms and showed that, following the stimulation of toll-like receptors, the autism group with GI symptoms have higher levels of cytokines such as interleukin-5 (IL-5) and interleukin-17 (IL-17) compared to the group without GI symptoms [5]. The production of regulatory cytokine-transforming growth factor beta-1 (TGFβ1) was lower in the autism group with GI symptoms [5]. The abundance of intestinal flora in children with ASD undergoes many changes between species. *Coprococcus*, *Akkermanis*, and *Ruminococcus *strains are increased in ASD children [6]. It has been proven that *Lactobacillus* and *Bifidobacterium* strains have anti-inflammatory properties, and they act by reducing interleukin-2 (IL-2), interferon-γ, interleukin-4 (IL-4), interleukin-13 (IL-13), and interleukin-17A (IL-17A), increasing anti-inflammatory cytokine interleukin-10 (IL-10) [7]. These cytokines have altered the serum of children with ASD, and chemokines also seem to play a role in ASD [8-10]. Studies have shown that *Collinsella* and *Clostridium *also increase in children with ASD [11,12]. This systematic review will provide a clearer and broader picture of probiotics’ effectiveness and safety profile. This review will also demonstrate different types and combinations of probiotic strains used in several studies, which will provide specifications of the most recent data.

## Review

Methods

The Preferred Reporting Items for Systematic Review and Meta-Analysis (PRISMA) 2020 guidelines were employed for performing this systematic review [13].

Search Sources and Search Strategy

Pubmed, Embase, PubMed Central (PMC), ScienceDirect, and Cochrane Library were searched to find studies published between 2017 and 2022. We began by exploring these databases by using three search keywords. We combined these keywords with the boolean operator “AND.” We used the following concept identification words for the keyword probiotics: prebiotics, *Bifidus*, and *Lactobacillus*. These words were joined by the boolean operator “OR,” and a medical subject heading (MeSH) search strategy was formulated. Similarly, for the keyword “gut microbiome,” the concept identification words used include microbiota, gut flora, and microflora. These words were also joined by “OR.” For the keyword “autism spectrum disorder,” the concept identification words used include autistic disorder, autistic, and pervasive developmental disorder. Again, these keywords were joined by “OR.” Finally, all MeSH search strategies were compiled using the boolean operator “AND,” which was then entered in PubMed. The following MeSH strategies were employed: “Probiotics/pharmacology”[MeSH] OR “Probiotics/physiology”[MeSH] OR “Probiotics/therapeutic use”[MeSH]) AND (“Brain-gut axis”[MeSH]) OR “Gastrointestinal microbiome”[MeSH] AND (“Autism spectrum disorder/etiology”[MeSH] OR “Autism spectrum disorder/therapy”[MeSH]) OR (“Autism spectrum disorder/diet therapy”[MeSH]).

Different keywords were used on databases. Similar combinations were used on other databases to extract relevant articles.

Screening

We started by screening and removing duplicate articles. Then, the remaining articles were screened based on titles or abstracts. Based on the quality analysis, full-text articles were included. Articles that were selected had to be published in English between 2017 and 2022. Articles included only human subjects, specifically those of the age group from birth to 18 years. 

Eligibility Criteria 

The inclusion criteria for this systematic review include human subjects from birth to 18 years and articles published in English, those specifically related to ASD, and those published from 2017 to 2022. A few papers were excluded because they focused on animal species or were published before 2017. The unpublished, irrelevant, or gray literature was also excluded. 

Results

Search Outcome

The quality of the papers included was assessed using the tools mentioned in Table 1, and only the articles that satisfied >60% of the appraisal parameters were considered (Table 1).

**Table 1:** Quality assessment using preferred tools. AMSTAR: Assessment of Multiple Systematic Reviews

Type of study	Tool used	Number of studies
Systematic review	AMSTAR Checklist	5
Randomized controlled trial	Cochrane Bias Assessment Tool	3
Non-randomized controlled trial	Newcastle-Ottawa Tool	1

**Figure 1:**
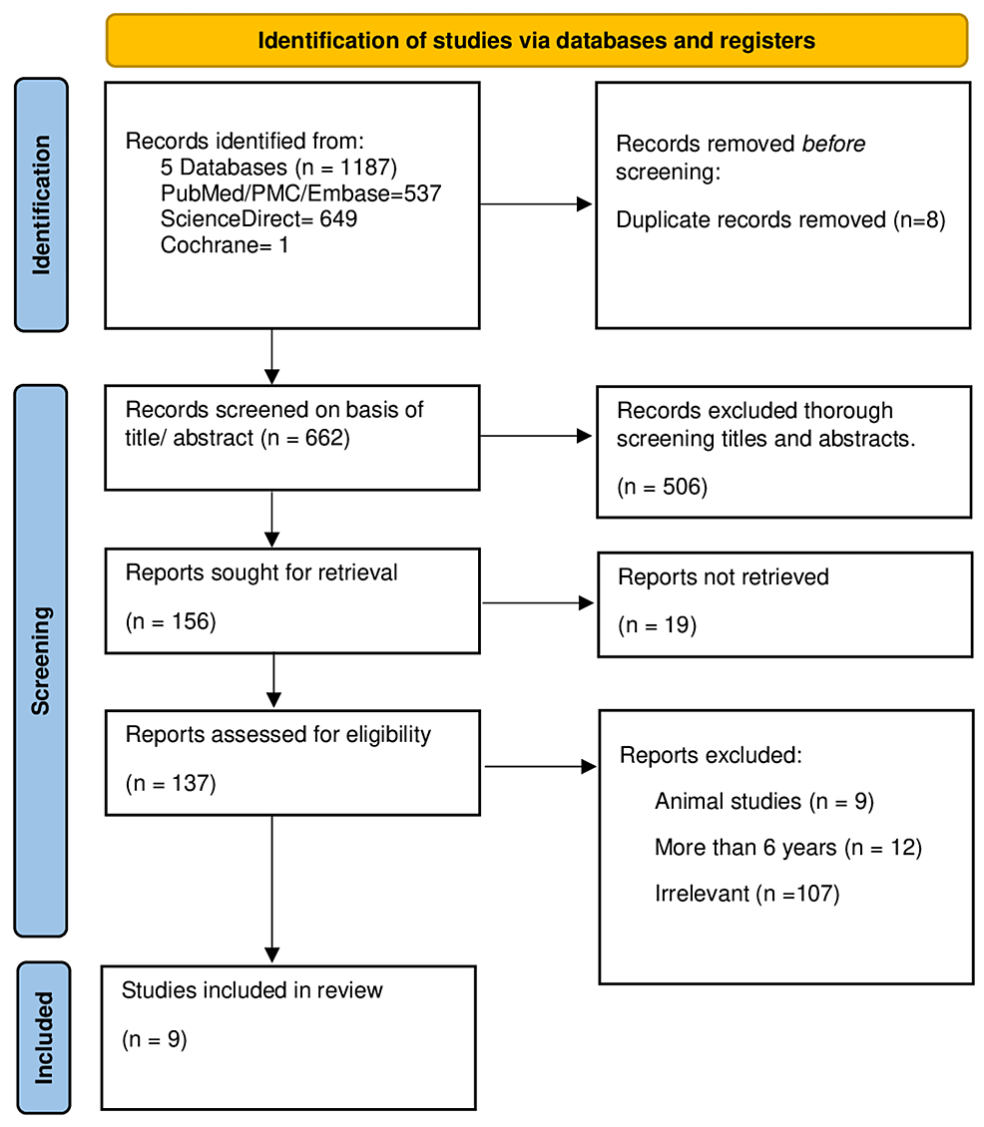
Preferred Reporting Items for Systematic Reviews and Meta-Analyses (PRISMA) flow diagram. PMC: PubMed Central

A total of 1,187 articles were identified using MeSH and keywords from PubMed, Embase, PMC, ScienceDirect, and Cochrane Library. Of these articles, eight duplicates were removed. After applying the inclusion/exclusion criteria in the initial stage, 662 articles were retained. After scanning the articles through titles and abstracts, 156 articles were obtained. A total of 137 papers were further assessed for eligibility. After full-text reading, 16 articles were found to have specific information related to the research topic. Quality appraisal of the 16 articles was done, of which 10 were included for review as they satisfied >60% of the appraisal parameter. Out of these articles, seven were systematic reviews, three were randomized controlled trials (RCTs), and one was a non-RCT. These articles mentioned the significance and effects of probiotics in children with ASD. The literature-screening process for the selection of eligible studies was performed based on the PRISMA guidelines, as shown below in Figure 1 [13].

Quality Appraisal Studies 

This systematic review included three types of studies: systematic review, RCT, and non-RCT and observational study. The quality of systematic reviews was assessed using the Assessment of Multiple Systematic Reviews (AMSTAR) Checklist. The findings are summarized below in Table 2*.*

**Table 2:** Quality appraisal of the systematic reviews. Key: + is Yes; - is No.

Included articles	Prior design performed	Duplication study - selection and abstraction	Comprehensive research search performed	Gray literature included	List of studies (inclusion and exclusion criteria used)	Characteristics of studies included
Yang et al. [14]	+	-	+	-	+	+
Salvatore et al. [15]	+	-	+	-	+	+
Song et al. [16]	+	-	+	-	+	+
Patusco et al. [17]	+	-	+	-	+	+
Ng et al. [18]	+	-	+	-	+	+

The quality appraisal for the RCT was performed using the Cochrane Bias Assessment Tool (Table 3).

**Table 3:** Quality appraisal for the randomized controlled trial. Key: + is Yes; - is No.

	Random sequence generation	Allocation concealment	Finding participants & personnel	Binding of outcome assessment	Incomplete outcome data	Selective reporting	Other bias
Sanctuary et al. [19]	+	+	+	+	-	-	Ascertainment bias
Arnold et al. [20]	+	+	+	+	-	+	Many dropped out

The quality appraisal of the non-RCT and observational study was done using the New-Castle Ottawa Tool. The results are shown below in Table 4. 

**Table 4:** Quality Appraisal for Non-Randomized Controlled Trial and Observational Study Article. Key: + is Yes,  -  is No

	Selectivity				Compatibility	Outcome		
	Exposed truly representative of average	Selection of non-exposed cohort	Exposure ascertained by secure record or interview	Demonstration of the outcome of interest not present initially	Study controls for other variables	Follow-up long enough for the outcome to occur	Complete follow-up of all subjects accounted for	Subject(s) lost to follow-up unlikely to introduce bias
Mensi et al. [21]	+	-	+	-	-	+	+	+

Study Characteristics

Our five systematic reviews included a total of 473 participants. The purpose of each systematic review was to target gut microbiota and check the efficacy and impact of probiotics on children with ASD symptoms, both GI and behavioral symptoms. Some strains of probiotics regulate intestinal sensory afferents and enteric nervous system (ENS) activity. Interventions such as prebiotics, probiotics, vitamin A, antibiotics, and fecal microbiota transplant (FMT) were beneficial. Studies showed that probiotics alleviated the symptoms of constipation and diarrhea. Patients who took *Lactobacillus Plantarum *psychobiotic128 (PS128) showed greater improvements and fewer side effects than those who took other probiotics, which were clinically significant. Out of five systematic reviews, three showed that probiotic use benefits children with ASD. One review suggested that, rather than giving prebiotics or probiotics individually, the combined treatment is more effective.

Out of the RCTs, one did not show a statistically significant outcome. The supplementation with probiotic Bifidobacterium Infantis and bovine colostrum product (BCP) improved the gut health in children with ASD and GI symptoms. Combination probiotic-BCP supplementation has yet to be studied in a controlled and systematic trial. 

The non-RCT and observational study showed an improvement in the global functioning of patients, who were described to have more attention, better communication skills, and improved personal autonomies. All articles suggested that no major side effects were seen, and the overall safety profile of probiotics is really good. The relevant information from all the articles reviewed is displayed below in Table 5. 

**Table 5:** Relevant information from all the articles. ASD: autism spectrum disorder; RCT: randomized controlled trial; BCP: bovine colostrum product; GI: gastrointestinal; ADOS-CSS: Autism Diagnostic Observation Schedule-Calibrated Severity Score; PS128*: *Psychobiotic128

Author and Year of Publication	Purpose of the study	Number of participants/ studies	Type Of Study	Conclusion	Limitation
Yang et al. [14]	To target gut microbiota through interventions to check the efficacy and impact on GI and behavioral symptoms among autism spectrum disorder patients	16	Systematic review	Different gut microbial-based interventions (i.e., prebiotic, probiotic, vitamin A supplementation, antibiotic, and fecal microbiota transplant) were suggested as useful for potential treatment.	To develop a meta-analysis, more research is needed to focus on a multi-disciplinary way to homogenize sample characterization.
Salvatore et al. [15]	To confirm efficacy-proven strains, dose, duration, and effects of probiotics on neuro gastroenterological disorders	188	Systematic review	Probiotics (*Bifidobacterium longum* 1714) attenuated the increase in cortisol levels; Bifidobacteria caused subtle improvements in hippocampus-dependent visuospatial memory performance, with no adverse effects in children with autism.	More human studies to see the effects on the brain-gut axis, microbiota, and specific probiotic strains are needed to check safety in larger study groups.
Song et al. 2022 [16]	To explore whether prebiotics and probiotics improve the severity of GI symptoms in children with autism and conduct a systematic review of RCTs, which has not been done before.	144	Systematic review	Probiotics or prebiotics did not significantly cause betterment of the severity of GI problems. Due to open-label experiments saying otherwise, future research is needed.	Only three clinically controlled studies, with a smaller sample, did not separately analyze the effects on the severity of symptoms, and extended periods of evaluation are needed.
Patusco et al. [17]	To provide clinicians a clearer picture and solve parental queries on how probiotics affect ASD children with GI symptoms. More than half of the studies evaluate change in behavior as well.	117	Systematic review	All studies showed improvement in stools. Despite the variety of species, strains, doses, and duration of probiotics, it consistently altered the fecal microbiota or urine metabolites significantly. Reduction in the severity of ASD symptoms (not all reached clinical significance). Few side effects	More research, including optimal species, strength, and duration of probiotic therapy, are needed for more precise interventions.
NG et al. [18]	To demonstrate if prebiotics and probiotics are efficacious in treating ASD symptoms in children	8	Systematic review	Prebiotics and probiotics showed limited efficacy in managing GI and behavioral symptoms. Mixed conclusions through RCTs	There were no standardized probiotic regimens, varying duration of treatments, or multiple variable strains with different concentrations of probiotics.
Sanctuary et al. [19]	To check the efficacy of the probiotic-BCP supplementation; to explore the changes in intestinal microbiota as well as supplement tolerability	8	Randomized controlled trial	Bovine colostrum product appears to be well-tolerated alone when combined with probiotic Bifidobacterium infantis Improvement of chronic GI symptoms in ASD with this intervention was unique.	Small sample size, high heterogeneity.
Arnold et al. [20]	To compare the effects of *visbiome* (eight probiotic species) if they improved quality of life in ASD	13	Randomized controlled trial	No major side effects; the *visbiome* formulation was safe and showed health benefits in patients with ASD concerning GI symptoms.	Larger trials using a parallel group design are still needed.
Mensi et al. [21]	Changes after probiotics (e.g., *Lactobacillus plantarum *PS128) consumption in ASD children through clinical global impression	131	Non-randomized controlled trial and observational study	Beneficial effects were seen, specifically with *Lactobacillus plantarum *PS128. Fewer side effects were seen.	Larger randomized prospective studies are still needed.
Santocchi et al. [22]	To evaluate ASD preschoolers with and without GI symptoms and the effects of the simple formulation regarding ASD core symptoms, GI symptoms, plasma, and fecal inflammatory biomarkers	85	Randomized controlled trial	Giving probiotic supplements did not give a statistically significant difference. However, for the first time in children without GI symptoms, significant modification of core ASD symptoms was seen by ADOS-CSS when treated with probiotics. (specifically social affect domain). It confirmed previous studies' data on few and transient side effects.	Large dropout rates. The use of ADOS-CSS evaluation as an outcome measure in clinical trials has been recently disputed, as it does not detect changes in the short term.

Discussion

Probiotics are defined as live microorganisms that, when administered in the right quantity, provide a benefit to an individual’s health. Microbial dysbiosis in autism is crucial to study if we want to find a cure.

Impact of Different Probiotic Strains on Restoring the Balance of the Gut Microbiome

The gut microbiota interacts significantly with the immune system. In children with ASD, immune dysregulation causes changes in circulating and brain cytokines and chemokines along with abnormal distributions and/or reactivity of leukocytes [23]. The association between the gut microbiome and the host’s immune system leads to homeostasis, while any interruption causes immune dysfunction [24]. One systematic review showed that probiotic supplements significantly altered the composition of the fecal microbiota and caused an increase in beneficial bacteria such as *Lactobacillus* and *Bifidobacteria *[25]. Other beneficial bacteria such as *Prevotella* and *Bacteroides* increased, while harmful bacteria such as *Firmicutes *and *Clostridium *decreased, which aligned with the outcomes of a previous meta-analysis [26]. *Clostridium* contributes to ASD through its potent neurotoxin effect, which inhibits neurotransmitter release [27]. Studies on the alteration of *desulfovibrio* are still controversial [28,29]. Exposure to antibiotics early in life may cause detrimental effects on the gut microbiota, which may cause recurring GI symptoms due to alterations in the microbiota composition [30]. Exposure to antibiotics early in life could be one of the possible causes of ASD [30]. However, this has yet to be studied [30]. It is still unclear which microbiota benefits or harms the homeostasis of the intestine [31]. *Lactobacillus Plantarum *PS128 is gram-positive, rod-shaped, facultative heterofermentative, and anaerobic, positively affecting children with ASD [32]. As seen in previous studies, there was a positive correlation between probiotics and younger age groups with a threshold around age 10, but the statistical analysis did not confirm this [32]. In summary, the exact mechanisms through which probiotics exert potential therapeutic effects have not yet been identified, but we understand that the gut microbiome plays a vital role. The down-regulation of inflammatory cytokines and effects on gut barrier permeability and immunomodulation are interlinked.

Influence Of Probiotics on Gastrointestinal Symptoms

The evidence shows that GI abnormalities and behavioral symptoms are characteristic in many children with ASD. One research supported a previous finding that gut dysbiosis is associated with the prevalence and severity of GI symptoms [33]. A study showed that combining prebiotics and probiotics might be more effective than taking either separately [21,34]. Several open-label experiments showed a reduction in GI problems on giving probiotic supplementation to children with ASD [35]. The study was conducted for a period of three months in a sample size of 30 ASD children between the ages of five and nine and showed that an effect was increased levels of *Bifidobacteria* in stool samples [35]. A similar outcome was seen in a randomized, double-blind, cross-over study done over three months that showed the decreased frequency of certain GI symptoms in both groups of patients (BCP only vs. BCP + *Bifidobacterium infantis*) [19]. Due to decreased IL-13 and Tumor Necrosis Factor-α (TNF-α) production, patients could have noted a decrease in symptoms [19]. In another randomized cross-over pilot trial study for *Visbiome* in ASD children from the age group of 3-12 years, the effect size, rather than statistical significance, was considered [20]. It showed that the first target symptom selected by parents showed improvement after eight weeks of probiotics compared to the placebo, but the second target symptom was not significant [20]. Thus, the average of these two could not show significance [20]. Most studies did not show any adverse effects as predicted [20]. *Visbiome* was considered a safe option, but we could not conclude if it helped improve the quality of life of ASD children [20]. Compared to other probiotics, *Lactobacillus plantarum* PS128 had greater improvements in the GI and core ASD symptoms and fewer side effects [21]. In summary, probiotics showed improvement in GI symptoms in the majority of our studies. The use of combined effects of probiotics and prebiotics seems controversial, and more studies are required in this area to determine if the combined effects are better than probiotics only. The side effects profile was safe in the majority of the studies.

Probiotics' Effects on Behavioral Symptoms

One study showed significant improvement in the stereotype and irritability scores in the group that received only BCP [19]. The reason could be decreased IL-13 and TNF-α production [19]. The scale they used to assess was the Behavior Rating Inventory of Executive Function and the Center for Epidemiological Studies Depression Scale for Children [21]. In contrast to this, one larger sample size, a two-center, randomized, double-blind, placebo-controlled study of 342 participants, showed contrary results [36]. When the mothers were given probiotics from 35 weeks of pregnancy until six months and children received the treatment from birth to two years, worse behaviors were noted after the treatment [36]. The one-label prospective study showed improved behavioral symptoms using the Autism Treatment Evaluation List [36]. One study mentioned the “opioid excess theory,” which holds that the breakdown products of dietary protein, especially casein and gluten, act as opioid receptor agonists to exert effects on the neurobehavioral aspect [37]. The main presumption of this theory is that autism is the result of a metabolic disorder [37]. Peptides with opioid activity derived from dietary sources enter the central nervous system through abnormally permeable intestinal membranes to affect neurotransmission [37]. Children with ASD developed several comorbidities, including diarrhea, constipation, commutative diarrhea/constipation, abdominal pain, vomiting, reflux, or bloating, which are positively correlated with the severity of behavioral symptoms [38]. In summary, children with ASD often develop gut-related comorbidities, and dysbiosis can negatively affect the GI tract and cause psychological symptoms. Both are interlinked. Probiotics seem to help with both GI and behavioral aspects.

Limitations

The literature consists of smaller studies with inconsistent measurements of GI symptoms. We need a larger sample size and homogenization of the characteristics of our sample to have a more interdisciplinary and collaborative approach. Changes in diet or intake of food that had probiotic sources were not controlled. Additionally, concurrent therapies and medications were not controlled. There were inconsistent strains, probiotic concentrates, and dosages, along with the probiotics’ duration. The small sample sizes, especially in studies related to the gut microbiota, could lead to inaccurate power that shows changes in participants. There were also variations in the psychological testing tools for testing the changes in behavioral aspects in children with ASD.

## Conclusions

This systematic review evaluated the outcome of probiotics as one of the interventions in children with ASD through their target on the intestinal microbiota. Children with ASD and GI symptoms experience impaired immune response due to gut microbiome dysfunction and bacterial dysbiosis. This study’s findings aligned with the existing data that the use of probiotics in children with ASD might be beneficial for both GI and psychological symptoms, especially with regard to certain specific probiotic strains. The combined use of prebiotics and probiotics seems to be beneficial. Despite the variability in species, doses, and duration of use, there was a consistent alteration in the fecal microbiota or urine metabolites in a beneficial way. It is possible that the effects of the probiotics in children with ASD, without having GI symptoms, may still be useful in treating the core symptoms. There are very few side effects to probiotics and the benefits outweigh the risks.
